# Positive clinical outcomes following therapy with programmed cell death protein 1/programmed cell death ligand 1 inhibitors in neuroendocrine carcinoma of the cervix

**DOI:** 10.3389/fphar.2022.1029598

**Published:** 2022-11-17

**Authors:** Rongyu Liu, Xinlin He, Zhengyu Li

**Affiliations:** ^1^ Department of Gynecology and Obstetrics, West China Second University Hospital, Sichuan University, Chengdu, China; ^2^ Key Laboratory of Birth Defects and Related Diseases of Women and Children (Sichuan University), Ministry of Education, Chengdu, China

**Keywords:** neuroendocrine carcinoma of the cervix, PD-1/PD-L1 inhibitors, immunotherapy, biomarkers, PD-L1 expression

## Abstract

Neuroendocrine carcinoma of the cervix (NECC) is a highly aggressive and rare gynecological malignancy with a poor prognosis. Despite aggressive local and systemic treatments, there are high rates of locoregional recurrence and distant metastases. Therefore, more potent treatments are required to manage NECC. In recent years, emerging immune checkpoint inhibitors, such as programmed cell death protein 1 (PD-1)/programmed cell death ligand 1 (PD-L1) inhibitors, have been used in treating various solid tumors and provide a new direction for immune-targeted therapy for NECC. In this review, we summarize the biomarkers useful for the evaluation of the therapy with PD-1/PD-L1 inhibitors in patients with NECC and the clinical applications and prospects of monotherapy with PD-1/PD-L1 inhibitors and combinations with other therapies in patients with NECC. In some individual case reports, therapeutic strategies with PD-1/PD-L1 inhibitors showed good efficacy. Further studies are needed to confirm the possibility of using PD-1/PD-L1 inhibitors as a standard treatment strategy in NECC.

## 1 Introduction

A range of tumors derived from neuroendocrine cells are known as neuroendocrine neoplasms (NENs). There is a high prevalence of NENs in the gastrointestinal tract, pancreas, and lungs. However, they rarely occur in the female genital tract. Neuroendocrine carcinoma of the cervix (NECC), which makes up only 1.4% of all cervical cancers, is a rare cervical neoplasm. (Tempfer et al., 2018). According to the most recent World Health Organization classification system for female genital tumors, NECC can be classified into two main cancer classes: poorly differentiated neuroendocrine carcinomas (NECs) and well-differentiated neuroendocrine tumors (NETs). Mitotic activity, nuclear atypia, and necrosis are used to classify NETs as grade 1 or 2 (McCluggage et al., 2022). Compared with NECs, NETs, including typical and atypical carcinoids, are rare in patients. Of these, the prevalence of NECs accounts for 92% of NECC patients. The prevalence of NETs is approximately 8% (Tempfer et al., 2018). According to cell morphology, NECs can be classified as either small-cell neuroendocrine carcinomas (SCNEC) or large-cell neuroendocrine carcinomas (LCNEC). Furthermore, there are mixed neuroendocrine-non neuroendocrine neoplasms (MiNENs). Among these, SCNEC is the most frequent type of NECC (Georgescu et al., 2021).

Human papillomavirus (HPV) is an important risk factor during the development of cervical cancer. NECC has been suggested in numerous studies as a possible HPV-related cancer as well. HPV can be found in virtually all cases of NECs (Kuji et al., 2017; Zhang et al., 2021). Castle et al. (2018) conducted a meta-analysis of the prevalence of any HPV type detected in 403 SCNEC and 45 LCNEC cases. They found that 85% of SCNEC and 88% of LCNEC cases were HPV positive, primarily HPV18 and HPV16. Several studies have suggested that HPV18 may be the most prevalent type in SCNEC (Pei et al., 2021; Takayanagi et al., 2021). Furthermore, some points of view suggested that HPV16 is more frequent in LCNEC, but there is insufficient evidence to support this view. The lack of evidence may be attributed to the low incidence of LCNEC, which makes large-scale studies difficult. In the future, extensive studies are needed to demonstrate this viewpoint. Most NECC are strongly and diffusely positive for p16 because of high-risk HPV, particularly HPV16 and HPV18 (Castle et al., 2018; Georgescu et al., 2021). Xing et al. (2018) performed an immunohistochemical evaluation of 10 SCNEC cases and found that all tumors exhibited diffuse/strong p16 expression. Alejo et al. (2018) confirmed the correlation between NECC and p16 overexpression. P16 immunohistochemical staining was performed in 44 cases in their study, 86% of which showed overexpression. Among these, all typical carcinoids, atypical carcinoids, and LCNEC were positive for p16, whereas 22 of 28 SCNEC (78.5%) were positive for p16. However, p16 positivity is of limited value in determining the site of origin, because neuroendocrine carcinomas arising at other sites may strongly express p16 due to a non-HPV-related process (Howitt et al., 2017; Xing et al., 2018). For example, a study showed that head and neck neuroendocrine carcinomas usually show strong, diffuse positive p16 immunostaining because of Rb pathway dysregulation. However, these tumors are rarely related to HPV infection (Alos et al., 2016).

NECC is characterized by high malignancy, distant metastasis, high mortality, and poor prognosis (Gadducci et al., 2017; Ishikawa et al., 2018; He et al., 2019). Compared with squamous cell carcinoma of the cervix (SCC) and cervical adenocarcinoma (ADC), NECC displays a highly aggressive biological behavior. It spreads mainly by lymphatic and blood metastases and is prone to lymphovascular space invasion and lymph node involvement (Gadducci et al., 2017). Local recurrence and distant metastasis can often occur (Zhang et al., 2021). Therefore, NECC carries a dismal prognosis and 5-year survival rates are relatively low (approximately 30%) compared to other forms of cervical cancer (Chen, J et al., 2021; Xu et al., 2018; Howitt et al., 2017; Gadducci et al., 2017; Ganesan et al., 2016; Margolis et al., 2016).

Therapeutic approaches to NECC represent a clinical challenge. Owing to its rarity, there are currently no standard therapeutic strategies based on prospective studies to against it. For NECC, treatment strategies are primarily borrowed from those for cervical cancer and small cell lung cancer (SCLC) (Chen, L et al., 2021). However, we identified several case reports using different therapeutic approaches for the treatment of NECC. All of them recommend a multimodal strategy based on systemic chemotherapy, radiation treatment, and surgery. In the early stages, radical hysterectomy and pelvic lymphadenectomy are usually performed, then adjuvant chemotherapy or concurrent chemoradiotherapy. Chemotherapy may have a significant beneficial effect on survival, and either carboplatin and etoposide or cisplatin and etoposide are the most commonly used chemotherapy regimens. If the tumor is limited to the cervix, therapies such as concurrent chemoradiation with brachytherapy can be used in combination with other systemic treatments. When the diameter of the tumor confined to the cervix is >4 cm, surgery or concurrent chemoradiotherapy can be performed after neoadjuvant chemotherapy. For locally advanced and metastatic disease, various strategies can be applied, including concurrent chemoradiation and brachytherapy with or without adjuvant chemotherapy, neoadjuvant chemotherapy followed by concurrent chemoradiation and brachytherapy, individualized external exposure combined with other treatments, systemic therapy, palliative support therapy, and pelvic exenteration. For patients with multiple recurrences of NECC, targeted therapy combined with chemotherapy may also play a beneficial role, which requires additional evidence for confirmation (NCCN Guidelines 2022; Zhang et al., 2021; Chen, J et al., 2021; Frumovitz et al., 2017; Gadducci et al., 2017; Burzawa et al., 2015).

Despite multimodal treatment, patients with NECC still carry a dismal prognosis, necessitating the urgent need for novel and efficient therapeutic approaches. The use of immune checkpoint inhibitors, such those of programmed cell death protein 1 (PD-1) and programmed cell death ligand 1 (PD-L1), have recently achieved breakthrough progress in the field of tumor treatment. The PD-1/PD-L1 signaling pathway is a key mechanism that facilitates tumor cell immune escape (Ngoi et al., 2018; Ramos-Casals et al., 2020). PD-1 is an immune checkpoint receptor mostly present on the surface of activated T lymphocytes. It mediates the negative regulatory signal of T-cell immunity and participates in the control of the tumor immune response in the negative direction (Cimic et al., 2021). PD-L1, the principal binding ligand of PD-1, is predominantly expressed in tumor cells and various immune cells (Li et al., 2018). An interacting PD-1 and PD-L1 not only inhibits the production of cytokines and T cell proliferation but also induces T cell apoptosis and transduces a negative signal, thus playing a negative regulatory role (Han et al., 2020). Some cancer therapies use monoclonal antibodies (those targeting PD-1 or PD-L1) to increase the immune system’s ability to combat tumor cells, causing the interaction between PD-1 and PD-L1 to be inhibited (Ramos-Casals et al., 2020). These monoclonal antibodies are known as PD-1/PD-L1 inhibitors. Owing to their broad antitumor applicability and durable antitumor effects, PD-1/PD-L1 inhibitors have become the most promising method of immunotherapy for cancer. The success of PD-1/PD-L1 inhibitors in treating cervical cancer and SCLC has further promoted research on their effect against NECC (Chen, L et al., 2021). This article reviews the current state and future prospects of PD-1/PD-L1 inhibitor treatment in NECC.

## 2 Predictive biomarkers of the efficacy of PD-1/PD-L1 inhibitors therapy in NECC

In recent years, PD-1/PD-L1 inhibitors have revolutionized the treatment of many types of cancers. They have also been applied to many gynecological malignancies, producing significant responses (Ngoi et al., 2018). It is worth investigating whether PD-1/PD-L1 inhibitors can be applied to NECC. To achieve precision therapy, it is particularly important to select specific biomarkers for screening populations that will benefit from treatment with PD-1/PD-L1 inhibitors, prior to treatment. We investigated the role of the PD-L1 expression, high microsatellite instability (MSI-H)/mismatch repair deficiency (dMMR), and tumor mutation burden (TMB) as predictive biomarkers of PD-1/PD-L1 inhibitors.

In clinical practice, PD-L1 expression is the most widely utilized predictive biomarker for assessing the response to PD-1/PD-L1 inhibitors. The immune escape of tumor cells can be promoted by PD-L1 overexpressed in tumor cells or tumor-infiltrating lymphocytes (TILs). Additionally, the efficacy of PD-1/PD-L1 inhibitors is significantly associated with PD-L1 overexpression (Chen, L et al., 2021). Therefore, PD-L1 is an important therapeutic target and tool for selecting candidate patients for immunotherapy and for predicting efficacy.

The DNA mismatch repair (MMR) system can fix mistakes that might happen when replicating DNA. Its deficiency leads to a buildup of mutations in coded and non-coded microsatellites. This phenomenon is called microsatellite instability (MSI) (Chen, L et al., 2021). MSI is categorized into three phenotypes: MSI-H, low microsatellite instability (MSI-L), and microsatellite stable (MSS). MSI-H or loss of MMR protein expression has been described as dMMR (Yamazaki et al., 2018). Owing to the failure of repairing DNA replication errors, MSI-H/dMMR tumors have an increased mutation rate and express high levels of neoantigens, making tumor cells immunogenic. Therefore, MSI-H/dMMR tumors respond well to PD-1/PD-L1 inhibitors (Le et al., 2017). TMB is expressed as the amount of nonsynonymous somatic mutations in the coding region of tumor cells. If it≥10 mutations per megabase (mut/Mb), it is considered a high tumor mutation burden (TMB-H) (Shiravand et al., 2022). TMB-H caused by MSI-H not only induces increased neoantigen expression and attracts more TILs, but also boosts PD-L1 expression, thus rendering tumor cells sensitive to PD-1/PD-L1 inhibitors (Chen, L et al., 2021). Notably, although MSI-H can lead to TMB-H, the role of TMB-H in forecasting the response to PD-1/PD-L1 inhibitors does not rely only on MSI-H. As MSI-H/dMMR and TMB status can effectively predict whether patients will benefit from receiving PD-1/PD-L1 inhibitor treatment, they can also be used as predictive factors.

Morgan et al. (2019) tested 10 samples collected from patients with SCNEC using immunohistochemistry (IHC). They discovered that 70% of SCNEC samples expressed PD-L1, predominantly focal, whereas 33% were characterized by a loss of MMR expression. PD-L1 expression was linked to the loss of MMR expression in more than 10% of tumor cells. Takayanagi et al. (2021) evaluated the expression of PD-L1 in samples collected from patients with NECC using IHC staining and found that 14 (56%) of 25 NECC samples were positive. Chen, L et al. (2021) used IHC to evaluate PD-L1 and MMR expression in 43 patients with SCNEC. Of the 43 patients, 22 (51%) had positive PD-L1 expression. All the patients were found to have MSS. Ji et al. (2021) performed IHC staining for assessment of PD-L1 and MMR expression on 20 NECC specimens. PD-1 was expressed in 14 cases (70%), while dMMR in six cases (30%). The results of these cohort studies indicate PD-1/PD-L1 inhibitors could be a feasible treatment option for patients with NECC.

Carroll et al. (2020) examined 40 pathological samples from patients with high-grade NECC. MSI testing was performed on 28 of the 40 samples, all of which were MSS. Further, 31 samples were examined for PD-L1 expression. Of 25 pure high-grade NECCs, only two (8%) were positive, while of six mixed tumors, three (50%) were positive. In the cohort study of Cimic et al. (2021), four (10%) of 39 cases of NECC were PD-L1 positive, and only one (3%) of 31 cases of NECC was TMB-H, and all 31 NECC cases were MSS. Their results indicated that 13% of patients with NECC could be candidates for therapy with PD-1/PD-L1 inhibitors. In these cohort studies, NECCs were classified as MSS without significant PD-L1 expression. Inhibitors of PD-1/PD-L1 may lack activity in these tumors.

As mentioned above, most patients with NECC were diagnosed with MSS in these studies. However, the PD-L1 expression in NECC patients varies across different studies. The various PD-L1 positive assessment criteria, tumor heterogeneity, and the limited patient sample may be to blame for these variances. In a study of SCNEC, more PD-L1 positive cases were found using RNA sequencing than IHC (36% vs 19%), suggesting that there may be more PD-L1 positive cases (Schultheis et al., 2015). Some patients with PD-L1-negative malignancies can nevertheless benefit significantly with PD-1/PD-L1 inhibitors, even though PD-L1 expression is a significant factor in the response to therapy. For example, nivolumab has a therapeutic impact on non-small cell lung cancer (NSCLC) patients who were PD-L1-negative (Horn et al., 2017). Balstilimab is effective for those with metastatic or recurring PD-L1-negative cervical cancer (O’Malley et al., 2021). There was also a case of PD-L1-negative SCNEC that responded completely to nivolumab, which will be mentioned below (Paraghamian et al., 2017). These cases support the possibility that PD-1/PD-L1 inhibitors could be useful in treating NECC. Although most NECC are MSS, other biomarkers such as TMB status can help predict how well a patient will react to PD-1/PD-L1 inhibitors. In the study of Eskander et al., 18 (18.6%) of 97 cases of high-grade NECC presented values of TMB higher or equal to 16 mut/Mb, but the number of cases with values of TMB higher or equal to 10 mut/Mb was not known (Eskander et al., 2020). Only few current studies on patients with NECC have measured TMB. Therefore, more research is required to determine the overall TMB status in NECC patients. Moreover, combining PD-1/PD-L1 inhibitors with other treatments, such as radiation therapy, ipilimumab, and poly (ADP-ribose) polymerase (PARP) inhibitors, may improve efficacy. In summary, using PD-1/PD-L1 inhibitors is a promising immunotherapy approach for patients with NECC. Using predictive markers such as PD-L1 expression, MSI-H/dMMR, and TMB status may improve the outcome of the therapy.

## 3 Monotherapy with PD-1/PD-L1 inhibitors

PD-1/PD-L1 inhibitors might provide new hope to patients with NECC refractory to conventional treatment strategies. Three PD-1 inhibitors, including nivolumab, pembrolizumab, and cemiplimab, as well as three PD-L1 inhibitors, including atezolizumab, durvalumab and avelumab, have been authorized by the United States Food and Drug Administration (FDA) (Vaddepally et al., 2020). As nivolumab and pembrolizumab have been put into clinical practice for a longer while and more studies exist on them, clinical studies on nivolumab and pembrolizumab are highlighted in this review.

### 3.1 Nivolumab

Nivolumab can selectively block the interaction of PD-1 with its ligands and promote antitumor immunity. Nivolumab is effective for treating NSCLC, melanoma, MSI-H/dMMR metastatic colorectal cancer (CRC), metastatic SCLC, renal cell carcinoma (RCC), and other cancers (Guo et al., 2017). According to the National Comprehensive Cancer Network (NCCN) guidelines, nivolumab should be taken into consideration as a second-line or subsequent treatment for PD-L1 positive recurrent or metastatic cervical malignancies (NCCN Guidelines 2022). According to a study by Paraghamian et al. (2017), nivolumab therapy produced a complete response in a patient with PD-L1-negative, metastatic, and recurrent SCNEC. A dosage of 3 mg/kg of nivolumab intravenously (IV) was administered to the patient every 2 weeks. After two dosages, radiographic imaging showed that all the target lesions were reduced in size. Treatment was discontinued after the sixth dosage. Three weeks later, PET/CT showed that both target and non-target lesions had completely resoluted (Table 1). These results imply that nivolumab may offer a potential treatment option for recurrent NECC.

**TABLE 1:** Individual case reports with positive outcomes observed following therapy with PD-1/PD-L1 inhibitors in NECC.

Author	Year	Diagnosis	Treatment	Outcome
Paraghamian et al	2017	Recurrent, metastatic, and PD-L1 positive SCNEC	Nivolumab 3 mg/kg IV every 2 weeks	PET/CT showed complete resolution of all target and non-target lesions
Sharabi et al	2017	Metastatic and chemotherapy-refractory stage IV LCNEC with MSI-H, TMB-H, and a low expression of PD-L1	Nivolumab 240 mg IV every 2 weeks and SBRT 500 cGy × 4 fractions	Near-complete systemic resolution of disease
Patel et al	2020	High-grade NECC	Nivolumab 240 mg IV every 2 weeks and ipilimumab 1 mg/kg IV every 6 weeks	PR
Paterniti et al	2021	Recurrent, metastatic, and PD-L1-negative SCNEC	Nivolumab 240 mg IV every 2 weeks and ipilimumab 1 mg/kg IV every 6 weeks	PET/CT showed no signs of disease
Ji et al	2021	Recurrent, PARP1 positive and PD-L1 positive SCNEC with MSI-H	Albumin-bound paclitaxel 400 mg every 3 weeks plus tislelizumab 200 mg every 3 weeks IV + oral administration of anlotinib 8 mg/day for 14 days	The condition was well controlled and the supraclavicular lymph nodes and retroperitoneal masses were significantly reduced

PD-L1, programmed cell death ligand 1; NECC, neuroendocrine carcinoma of the cervix; SCNEC, small cell neuroendocrine carcinomas; LCNEC, large cell neuroendocrine carcinomas; MSI-H, high microsatellite instability; TMB-H, high tumor mutation burden; PARP1, poly (ADP-ribose) polymerase; IV, intravenous injection; cGy, centigray; PET/CT, Positron Emission Tomography/Computed Tomography; SBRT, stereotactic body radiation therapy; PR, partial remission.

### 3.2 Pembrolizumab

Pembrolizumab can bind to PD-1, antagonize PD-1 interaction with its ligands, and enable the immune system to eliminate cancer cells (Sahni et al., 2018). The first tissue-agnostic/site-agnostic medication, pembrolizumab is effective for treating melanoma, NSCLC, recurrent or metastatic cervical cancer, metastatic SCLC, endometrial carcinoma, TMB-H solid tumors, MSI-H or dMMR CRC, triple-negative breast cancer expressing PD-L1 and other types of cancer (Arias-Pinilla and Modjtahedi 2021). In addition, the NCCN guidelines state pembrolizumab can be considered a second-line or subsequent treatment for cervical malignancies that are PD-L1 positive, MSI-H/dMMR, and TMB-H recurrent or metastatic (NCCN Guidelines 2022). In the clinical trial of Frumovitz et al, (2020), none of the six SCNEC cases responded to monotherapy with pembrolizumab. However, the small sample size is not sufficient to deny the efficacy of pembrolizumab against NECC. The efficacy of pembrolizumab may increase when used in conjunction with other therapies, such as standard chemotherapy or PARP inhibitors. There is a need for a higher level of evidence from randomized clinical trials to confirm the efficacy and safety of pembrolizumab in treating NECC.

## 4 Promising combination therapy strategies

Comprehensive therapy that integrates multiple treatment methods is the future development direction for cancer treatment. Combining conventional therapies with PD-1/PD-L1 inhibitors can result in substantial synergism that may significantly improve clinical outcomes. Hence, next, we discuss the clinical studies using PD-1/PD-L1 inhibitors in conjunction with other therapies for treating patients with NECC.

### 4.1 PD-1 inhibitors combined with radiotherapy

Preclinical studies have reported that immunotherapy + radiotherapy can produce a significant response called the “abscopal effect”. The abscopal effect refers to the clinical phenomenon in which local irradiation may cause distant tumors in the non-irradiated area to regress (Weichselbaum et al., 2017). This is because radiotherapy can cause the death of tumor cells, causing the release of tumor-specific antigens. Then, through the antigen cross-presentation of antigen-presenting cells (dendritic cells and macrophages) around the tumor, cytotoxic T cells against these antigens can be activated. These cytotoxic T cells can spread through the circulatory system to the whole body and kill distant metastatic tumor cells far from the irradiated area. In addition, tumor cells killed by radiotherapy may release cytokines and damage-associated molecular patterns (DAMPs) to promote immune responses against tumors (Shan et al., 2021). Compared to PD-1 inhibitors or stereotactic body radiation therapy (SBRT) alone, combining a PD-1 inhibitor with SBRT may have synergistic antitumor effects on the primary tumor (Gong et al., 2018). In a variety of tumor models, combining radiotherapy with PD-1 inhibitors resulted in longer survival and other synergistic benefits (Zeng et al., 2013; Sharabi et al., 2015). Preclinical research indicates that combining radiotherapy with PD-1/PD-L1 inhibitors may enhance anticancer activity by increasing interferon γ, tumor antigen cross-presentation, T-cell receptor clonality, PD-L1 expression, and reinvigorating TILs. This combination could result in fewer regulatory T cells and myeloid-derived suppressor cells.

Sharabi et al. (2017) reported a patient with stage IV LCNEC that developed rapidly during radiotherapy and chemotherapy. The patient developed a partial ileus owing to a large tumor burden. Moreover, tissue genomic results showed MMR alterations (MSH2 gene), TMB-H (53mut/Mb), and MSI-H status. IHC revealed low PD-L1 expression. A dosage of 240 mg of IV nivolumab was given to the patient every 2 weeks, and SBRT was given (500 centigray × 4 fractions) 2 weeks after the initiation of immunotherapy. At 2 months, the patient showed excellent partial remission (PR), and at 6 months, had a nearly full response. The response persisted after 11 months and over 95% of the tumor had regressed (Table 1). This case report indicates that advanced NECC may benefit from a combination of SBRT and PD-1 inhibitors, and this combination could be a successful therapeutic approach for NECC patients with low PD-L1 expression.

### 4.2 PD-1 inhibitors combined with ipilimumab

By preventing CTLA-4 from attaching to its ligands (CD80 and CD86), ipilimumab can promote activation and proliferation of T cells and take part in the immune response to tumors. Ipilimumab is licensed to treat metastatic or unresectable melanoma as well as to be given to patients with stage III melanoma as adjuvant treatment. It has also been approved for use in conjunction with nivolumab to treat PD-L1 positive metastatic NSCLC, MSI-H, or dMMR metastatic CRC, and other cancers (Arias-Pinilla and Modjtahedi 2021).

Thirty-two patients with nonpancreatic neuroendocrine tumors, three of whom had high-grade NECC, were included in a phase II basket trial using dual therapy with anti-CTLA-4 and anti-PD-1 inhibitors in treating uncommon cancers (DART SWOG 1609). The treatment regimen was comprised of ipilimumab 1 mg/kg IV every 6 weeks and nivolumab 240 mg IV every 2 weeks. The results of this trial showed that the overall response rate of patients with high-grade NECs was 44% (8/18patients); of them, one patient with NECC achieved PR (Patel et al., 2020) (Table 1). This indicated the feasibility of this combination therapy for NECC. Nivolumab plus ipilimumab treatment resulted in a full and long-lasting response in a recurrent, metastatic, PD-L1-negative SCNEC case, according to Paterniti et al. (2021). The patient presented liver, lung, and brain metastasis. Over the course of a decade, she received radical surgical treatment, six types of systemic chemotherapy, and radiotherapy to the pelvis and brain. After the second relapse, nivolumab 240 mg IV every 2 weeks and ipilimumab 1 mg/kg IV every 6 weeks were given to the patient. The patient continued receiving nivolumab and ipilimumab therapy a year after beginning treatment, and PET-CT showed no signs of disease (Table 1). According to this case report, nivolumab + ipilimumab may be a viable new therapy option for those with recurrent SCNEC who are not responding to standard care.

### 4.3 PD-1/PD-L1 inhibitors combined with PARP inhibitors

PARP is a DNA repair enzyme that performs an essential function in the restoration of DNA damage. It is also considered an important indicator of apoptosis. By inhibiting DNA damage repair and promoting apoptosis of tumor cells, PARP inhibitors can be used for cancer therapy (Lord and Ashworth 2017). In breast cancer cell lines and animal models, Jiao et al. (2017) demonstrated that PD-L1 expression can be upregulated by PARP inhibitors. PARP inhibitors increase the resistance of tumor cells to T cell-mediated cell death by upregulating PD-L1, whereas blocking PD-L1 can make cancer cells that have been treated with PARP inhibitors more susceptible to T cell killing. According to a study by Sen et al. (2019), PARP inhibitors dramatically increased PD-L1 expression and remarkably potentiated the anticancer impact of PD-L1 blockade in the SCLC mouse model. Moreover, the combination of PARP inhibitors and PD-L1 blockade can not only cause remarkable tumor regression but can also increase CD8^+^ cytotoxic T cells infiltration and reduce the levels of exhausted and regulatory T cells. These studies indicate that the combination of PD-1/PD-L1 inhibitors and PARP inhibitors has multiple synergistic effects that are superior to those of either monotherapy.

Rose et al. (2019) reported a patient with stage IV NECC with a BRCA mutation. She received oral rucaparib 600 mg twice a day and was progression-free for 15 months. Carroll et al. (2020) tested the expression of PARP-1 in 11 SCNEC specimens. Of these, ten (91%) were PARP positive, with four (36%) having moderate levels of expression, and six (55%) high levels of expression. The expression of PARP-1 in the majority of the tumors tested may indicate that NECC responds favorably to PARP inhibitors. However, no reports exist on the use of PARP inhibitors combined with PD-1 /PD-L1 inhibitors against NECC; therefore, such studies are needed. Although PD-L1 expression is negative in some NECC patients, as mentioned above, PARP inhibitors may induce PD-L1 expression, thus leading to greater therapeutic benefits. In summary, combining PD-1/PD-L1 inhibitors with PARP inhibitors may be a viable treatment option for NECC.

### 4.4 Other combined therapies

Individualized treatment plans are possible to treat this aggressive tumor when one or more mutated genes in NECC that respond to targeted therapy are present. Genetic alterations that affect the MAPK, PI3K/AKT/mTOR, and TP53/BRCA pathways in SCNEC were validated by Xing et al. using targeted next-generation gene sequencing (Xing et al., 2018). Hillman et al. (2020) performed whole exome sequencing in 15 cases of high-grade NECC tissue. Of these, PI3-kinase or MAPK activating mutations, including PIK3CA activating mutations, KRAS/GNAS activating mutations and PTEN loss were found in 67% of tumors. For NECC with multiple recurrences, a combination of targeted therapies can bring positive efficacy. Currently, targeted therapies for patients with NECC lack sufficient knowledge and applications, necessitating further research and development.

The combination of PD-1/PD-L1 inhibitors with chemotherapy and/or anti-angiogenic drugs may result in new treatment options for patients with NECC. Chemotherapy can induce cancer cell death, promoting tumor antigen release, increasing antigen presentation, and stimulating immune effectors, which may enhance antitumor immunity. PD-1/PD-L1 inhibitors + chemotherapy have a magnificent efficacy, which can be used in upper gastrointestinal malignancies, triple-negative breast cancer, NSCLC, SCLC, and other cancers (Paz-Ares et al., 2018; Cortes et al., 2020; Zhang et al., 2020; Janjigian et al., 2021). In Tangjitgamaol et al.’s study, 23 (96%) of 24 patients with NECC expressed vascular endothelial growth factor (VEGF) (Tangjitgamaol et al., 2005). Within the tumor microenvironment, angiogenesis driven by VEGF is considered a critical factor of tumor-induced immunosuppression. The combination of anti-angiogenic drugs with PD-1/PD-L1 inhibitors is synergistic as it can not only attenuate tumor-induced immunosuppression but also promote antitumor immunity and normalization of tumor blood vessels (Fukumura et al., 2018; Hack et al., 2020). This combination has been authorized by the FDA for RCC, endometrial carcinoma, NSCLC, and hepatocellular carcinoma (Hack et al., 2020). Moreover, the PD-1 inhibitor pembrolizumab, combined with chemotherapy and anti-angiogenesis drugs, has also been approved by the FDA as a first-line therapy for ongoing, recurring, or metastatic cervical cancer in which the tumors express PD-L1 positivity (Colombo et al., 2021). Ji et al. (2021) reported a patient with MSI-H SCNEC and PD-L1 positivity. The patient was treated with chemotherapy after the second relapse; however, the disease progressed during chemotherapy. After 5 months, the treatment plan was changed to include oral anlotinib (8 mg/day) for 14 days, albumin-bound paclitaxel 400 mg every 3 weeks, and tislelizumab 200 mg every 3 weeks. After a treatment period, the patient’s condition was well controlled (Table 1). As a result of this comprehensive treatment strategy, a synergistic effect may be achieved and tumor immune tolerance may be reversed. Additional studies must be conducted in order to determine whether PD-1/PD-L1 inhibitors are safe and efficient in conjunction with chemotherapy and/or antiangiogenic drugs in the treatment of NECC.

## 5 Conclusion

In recent years, significant advancements have been made in research on PD-1/PD-L1 inhibitors and their combination therapies. Several studies have examined biomarkers in NECC patients, suggesting that some NECC patients may benefit from receiving PD-1/PD-L1 inhibitor treatment. Owing to the rarity of NECC, it is difficult to conduct special trials to confirm the efficacy of PD-1/PD-L1 inhibitors in treating this disease. Therefore, evidence obtained from any individual case study is critical to facilitate further research and practice. The currently reported individual cases indicate positive clinical outcomes with nivolumab monotherapy, the combination of nivolumab and SBRT or ipilimumab, and tislelizumab combined with albumin-bound paclitaxel and anlotinib. In addition, other therapies, such as pembrolizumab and the combination of PD-1/PD-L1 inhibitors and PARP inhibitors, also have the potential to be effective against NECC. However, their efficacy should be further confirmed. Furthermore, it is worth exploring the efficacy of other therapeutic strategies against NECC: PD-L1 inhibitors and the combination of PD-1/PD-L1 inhibitors with therapeutic cancer vaccines, epidermal growth factor receptor tyrosine kinase inhibitors (EGFR-TKIs), or other targeted therapies. Analyzing predictive biomarkers to identify NECC patients who will benefit most from monotherapy with PD-1/PD-L1 inhibitors or combination treatment and tailoring individualized treatment regimens specific to different situations may greatly improve patient outcomes. Large-scale prospective and multi-institutional studies are needed to determine whether PD-1/PD-L1 inhibitor monotherapy and in conjunction with other therapies can be used as a novel standard treatment strategy for NECC.

## Glossary

ADC: Cervical adenocarcinoma
BRCA: Breast cancer gene
CPS: Combined positive score
CRC: Colorectal cancer
CTLA: Cytotoxic T lymphocyte-associated protein
dMMR: Mismatch repair deficiency
DAMP: Damage-associated molecular patterns
EGFR-TKI: Epidermal growth factor receptor tyrosine kinase inhibitor
FDA: The Food and Drug Administration
GNAS: Guanine nucleotide binding protein, alpha stimulating
HPV: Human papillomavirus
IHC: Immunohistochemistry
IV: Intravenously
KRAS: Kirsten rat sarcoma viral oncogene homolog
LCNEC: Large cell neuroendocrine carcinomas
MAPK: Mitogen-activated protein kinase
MiNENS: Mixed neuroendocrine-non neuroendocrine neoplasms
MMR: Mismatch repair
MSH2: MutS protein homolog 2
MSI: Microsatellite instability
MSI-H: High microsatellite instability
MSI-L: Low microsatellite instability
MSS: Microsatellite stable
mTOR: Mammalian target of rapamycin
NCCN: National Comprehensive Cancer Network
NECC: Neuroendocrine carcinoma of the cervix
NECs: Neuroendocrine carcinomas
NENs: Neuroendocrine neoplasms
NETs: Neuroendocrine tumors
NSCLC: Non-small cell lung cancer
PARP: Poly (ADP-ribose) polymerase
PD-1: Programmed cell death protein 1
PD-L1: Programmed cell death ligand 1
PET/CT: Positron emission tomography/computed tomography
PI3K: Phosphoinositide 3-Kinase
PIK3CA: Phosphatidylinositol-4,5-bisphosphate 3-kinase catalytic subunit alpha
PR: Partial remission
PTEN: Phosphatase and tensin homolog
RCC: Renal cell carcinoma
SBRT: Stereotactic body radiation therapy
SCC: Squamous cell carcinoma of cervix
SCLC: Small cell lung cancer
SCNEC: Small cell neuroendocrine carcinomas
TILs: Tumor-infiltrated lymphocytes
TMB: Tumor mutation burden
TMB-H: High tumor mutation burden
VEGF: Vascular endothelial growth factor
